# Intravitreal fluocinolone acetonide 0.19 mg (ILUVIEN®) in patients with non-infectious uveitis: real-world effectiveness and safety outcomes at 12 months

**DOI:** 10.1007/s10792-023-02828-6

**Published:** 2023-09-12

**Authors:** Aina Moll-Udina, Inés Hernanz, Maite Sainz-de-la-Maza, Laura Pelegrín, Ana Isabel Coelho-Borges, Marta Pazos, Alfredo Adán, Victor Llorenç

**Affiliations:** 1 Facultat de Medicina, University of Barcelona, Barcelona, Spain; 2 Clínic Institute of Ophthalmology, Clínic Hospital of Barcelona, Sabino de Arana Str., 1, 2Nd Floor (Maternity Hospital), 08028 Barcelona, PC Spain; 3 August Pi I Sunyer Biomedical Research Institute (IDIBAPS), Barcelona, Spain; 4 Hospital Fundacion Jiménez Díaz, Madrid, Spain; 5 CIICESI, ESTG, Politécnico Do Porto, Porto, Portugal

**Keywords:** Non-infectious uveitis, Macular oedema, Fluocinolone acetonide implant, Real-world effectiveness, Intraocular inflammation

## Abstract

**Purpose:**

This study assessed the effectiveness of the 0.19-mg fluocinolone acetonide (FAc) implant by multimodal measurements in patients with non-infectious uveitis (NIU) in a real-world setting in Spain.

**Methods:**

A prospective study of patients who had NIU including uveitic macular oedema (UME) with ≥ 12 months follow-up was done. Exclusion criteria include infectious uveitis and uncontrolled glaucoma or ocular hypertension requiring more than 2 medications. Effectiveness was assessed using a multicomponent outcome measure that included nine outcomes. Effectiveness was defined as all components being met at every timepoint. Secondary outcome measures were onset or progression of glaucoma and investigator-reported adverse events.

**Results:**

Twenty-six eyes from 22 patients were included, with 96.2% having an indication including UME. During the 12-month study, the FAc implant was effective in 15 (57.7%) eyes, reaching effectiveness as soon as 2 weeks post-implantation. Mean best-corrected visual acuity and mean central macular thickness (CMT) were significantly improved vs. baseline at all timepoints (all comparisons *p* < 0.01). During the 12-month study, inflammation markers (anterior chamber cells and vitreous haze) had also significantly declined. Factors predicting effectiveness at month 12 were systemic corticosteroid dose pre-FAc, higher immunomodulatory therapy (IMT) load at baseline and thicker retinal nerve fibre layer (RNFL) at baseline (all *p* < 0.05). Factors predicting failure were male gender, thinner RNFL at baseline and treatment ineffectiveness at 1 month (all *p* < 0.05). In parallel, corticosteroid and IMT use also declined significantly. No significant increase in IOP was detected.

**Conclusion:**

The FAc implant is safe and effective at treating NIU over 12 months in a real-world setting in Spain.

## Introduction

Non-infectious uveitis (NIU) is a clinically heterogenous group of inflammatory disorders of the eye responsible for ~ 15% of adult visual impairment in the developed world [1]. Often affecting individuals of working age, NIU is associated with a substantial socioeconomic impact in terms of both direct (e.g. clinic visits and treatment) and indirect costs (e.g. productivity loss due to impaired vision) [1, 2]. Currently, in the absence of a conclusive therapy, patients with NIU often require long-term care to manage their condition.

Macular oedema (ME)—defined as macular thickening due to fluid accumulation—can be a complication of uveitis. It is a leading cause of visual impairment (quantity and quality loss of central vision) [3, 4] which is found in approximately 40–44% of patients with uveitis [5, 6]. ME is the result of the breakdown of the outer and/or inner blood–retina barrier(s) caused by inflammatory mediators [4].

Despite progress in recent years, the pathophysiological mechanisms behind NIU and associated ME (termed uveitic macular oedema [UME]) are poorly understood, yet are known to be dependent on an array of inflammatory pathways [7, 8]. Chronic inflammation leads to structural and functional changes of the eye, a progressive ocular tissue damage and, ultimately, visual impairment [8, 9].

Targeting and controlling chronic inflammation in NIU is the basis for new treatment approaches [8]. Disease-modifying anti-rheumatic drugs (DMARDs), topical and periocular corticosteroids are all used in the treatment of NIU with variable success rates and heterogeneous side effects [10–12]. Systemic corticosteroids can be effective but are associated with ocular side effects (glaucoma, cataracts and ocular hypertension) and, as with DMARDs, systemic side effects (hypertension, diabetes, osteoporosis, gastrointestinal disturbances, etc.) when given in moderate–high doses and/or in a long-term period [4, 13].

A post hoc analysis from the VISUAL-1 and VISUAL-2 studies suggested that the incidence rates of corticosteroid-related adverse events (AEs) increase systematically with corticosteroid dose [14]. And, despite systemic treatments being effective in inflammation control, UME can persist in half of patients [5].

An alternative to these approaches is the implantation of intraocular sustained-release corticosteroid implants, which minimize the risk of systemic side effects. Moreover, intravitreal treatment can be a good option when systemic immunomodulatory therapy is contraindicated (e.g. recent cancer and severe osteoporosis).

In this setting, the 0.70-mg dexamethasone implant (Ozurdex® [DEX]) is associated with a significant gain in best-corrected visual acuity (BCVA [log minimum angle of resolution]) vs. sham injection, that is maintained up to 26 weeks [15]. Additionally, greater improvement in BCVA and central macular thickness (CMT) were noted with DEX compared to periocular triamcinolone up to 24 weeks [12]. However, UME relapses after 4–6 months of DEX implantation were frequent, leading to reinjections [16, 17], structural macular damage and visual acuity oscillations over time. Long-term effectiveness (up to 30 months) can be obtained with the 0.59- mg fluocinolone acetonide (FAc) implant (Retisert®). However, this implant is not approved in Europe due to the high risk of ocular complications, unlike in the USA [18]. Ocular hypertension, glaucoma and cataract surgery have been described [19].

Conversely, the 0.19-mg FAc implant (ILLUVIEN®)—which is an intravitreal, non-bioerodible implant that releases the drug steadily and continuously into the vitreous cavity for up to 3 years—is licensed in Europe for NIU affecting the posterior segment (NIU-PS) of the eye [20].

A Phase 3, prospective study comparing the 0.19-mg FAc implant to sham injections in NIU-PS demonstrated encouraging results, such as lower rates of recurrence, time to first recurrence and number of recurrences per eye and greater and more stable improvements in BCVA [21].

Herein, we describe the effectiveness of the 0.19-mg FAc implant in patients treated for NIU over ≥ 12 months using multimodal measurements.

## Materials and methods

### Study design

A prospective, 2-year study of NIU cases treated with the 0.19-mg FAc implant from November 2018 to November 2020 in a single referral uveitis unit in Spain (Clinic Hospital of Barcelona). The study was approved by the institutional review board (HCB/0440) and followed the tenets of the Declaration of Helsinki with all patients providing written informed consent.

### Patients

Patients were included if they were ≥ 18 years, had NIU (intermediate, posterior, panuveitis or anterior–intermediate uveitis) affecting the posterior segment of the eye—including macular oedema as activity criteria—, had ≥ 12 months follow-up and provided informed consent. The Standardization of Uveitis Nomenclature (SUN) Working Group recommendations were used to anatomically classify and grade each case [22]. Inflammatory activity as per vitreous haze (VH) score was based on the National Eye Institute (NEI) grading scale [23]. Exclusion criteria were infectious uveitis, uncontrolled glaucoma or ocular hypertension requiring more than 2 medications, low-quality optical coherence tomography imaging (Q < 7/10) and pregnant or breastfeeding women.

OCT scans (spectral-domain OCT; Cirrus HD-OCT®, Carl Zeiss Meditec, California, USA) were obtained in all patients after pupillary dilation. CMT, macular volume (MV), retinal nerve fibre layer (RNFL) and vertical cup/disc ratio data were determined automatically by the manufacturer’s built-on software.

### Outcomes

Effectiveness was assessed at week 2 and months 1, 3, 6 and 12 using a multicomponent outcome measure that included: BCVA (log minimum angle of resolution [LogMAR]) ≥ baseline; anterior chamber cells (ACC) (SUN) ≤ 0.5 + ; VH (NEI) ≤ 0.5 + ; no active chorioretinal or vascular lesions; CMT < baseline; immunomodulatory therapy score (IMTS) ≤ baseline based in Nussenblatt score [24]; oral prednisone or equivalent ≤ 7.5 mg/d; no new-onset or dosage increase in IMT and no adjuvant intravitreal therapy (IVT). The FAc implant was defined as effective if all components met at every timepoint; correspondingly, patients failed when any of the components did not meet at any timepoint.

Secondary outcome measures were survival of FAc until the first failure and, due to a potential incomplete effect at 2 weeks, the survival of FAc until the first failure after 2 weeks of injection. Regarding safety outcomes: onset or progression of glaucoma (depends on IOP, RNFL, vertical cup/disc ratio, fundus image of optic disc and visual field testing when necessary) recorded at any timepoint, along with final evaluation by a glaucoma specialist; investigator-reported adverse events (AE) at any timepoint.

### Statistical analyses

IBM SPSS V.28 was used for statistical analyses. McNemar’s test was used to analyse paired categorical data; for other data, the non-parametric signed-rank test, independent mean t-test and Mann–Whitney median test for comparisons were used. To predict treatment failure, a multivariate general estimating equation (GEE) model was applied at patient level to control a possible bias due to repeated measurements of both eye inclusion in the analysis, with logit link function, binomial distribution and an independent correlation matrix structure. The Kaplan–Meier method was used to estimate the survival rate of FAc until failure. For all tests, *p* < 0.05 was considered statistically significant.

## Results

### Patients

A total of 26 eyes from 22 patients were included. Patient demographics and baseline characteristics are shown in Table 1. FAc implant was injected in clinically active eyes, including relapsed UME in 96%, vitreous haze in 23%, optic disc swelling in 15% and anterior chamber cells in 27% of the eyes at the time of the injection. To remark, 10 (45.5%) patients suffered from an underlying condition (recent cancer, psychiatric disorders, severe osteoporosis and gastro-duodenal perforated ulcer) limiting systemic treatment with immunomodulators, including systemic corticosteroids (SCS). During the 12 months prior to FAc, a mean of 2 ± 0.86 DEX or triamcinolone acetonide injections per eye was used in 16/26 (62%) of the eyes. A local or systemic corticosteroid booster to reach quiescence prior to FAc implantation was not used in any eye in this study.

**Table 1:** Patient demographics and baseline characteristics

Characteristics	*N* = 26 eyes (22 patients)
*Age, years*	
Mean ± SD	65.0 ± 14.4
Range	33–90
Sex, % female	69.2%
Underlying condition limiting systemic therapy, n (%)	10 (38.6%)
*Single functioning eye, n (%)*	4 (15.4%)
Yes	
Duration of uveitis, months	
Mean ± SD	69.5 ± 58.4
Range	6–216
*Type of uveitis, n (%)*	
Anterior–intermediate uveitis	7 (26.9%)
Intermediate uveitis	7 (26.9%)
Posterior uveitis	6 (23.1%)
Panuveitis	6 (23.1%)
*Diagnosis of uveitis, n (%)*	
BCG HLA-B27 +	1 (3.8%)
Sarcoidosis	4 (15.4%)
Sympathetic ophthalmia	2 (7.7%)
Birdshot chorioretinitis	3 (11.5%)
Post-surgical uveitis	4 (15.4%)
Tubulointerstitial nephritis and uveitis	1 (3.8%)
IRVAN	1 (3.8%)
Blau syndrome-associated uveitis	2 (7.7%)
Unclassified	8 (31.0%)
Endogenous uveitis, n (%)	21 (81%)
Affected bilaterally, n (%)	17 (65.4%)
Corneal thickness, µm	
*Mean ± SD*	557 ± 38.2
Range	485–609
Prior glaucoma surgery, n (%)	2 (7.7%)
Lens status, pseudophakic, n (%)	23 (88.5%)
*Indication for FAc implant, n (%)*	
Central macular oedema (isolated)	18 (69.2%)
*Centra macular oedema in association with:*	
Vitreous haze	3 (11.5%)
Optic disc swelling	2 (7.7%)
Vitreous haze + optic disc swelling	2 (7.7%)
Vitreous haze (isolated)	1 (3.8%)

BCG, bacillus Calmette–Guérin; HLA, human leucocyte antigen; FAc, fluocinolone acetonide; IRVAN, idiopathic retinitis, vasculitis, aneurysms and neuroretinitis and SD, standard deviation

### Effectiveness

**Fig. 1:**
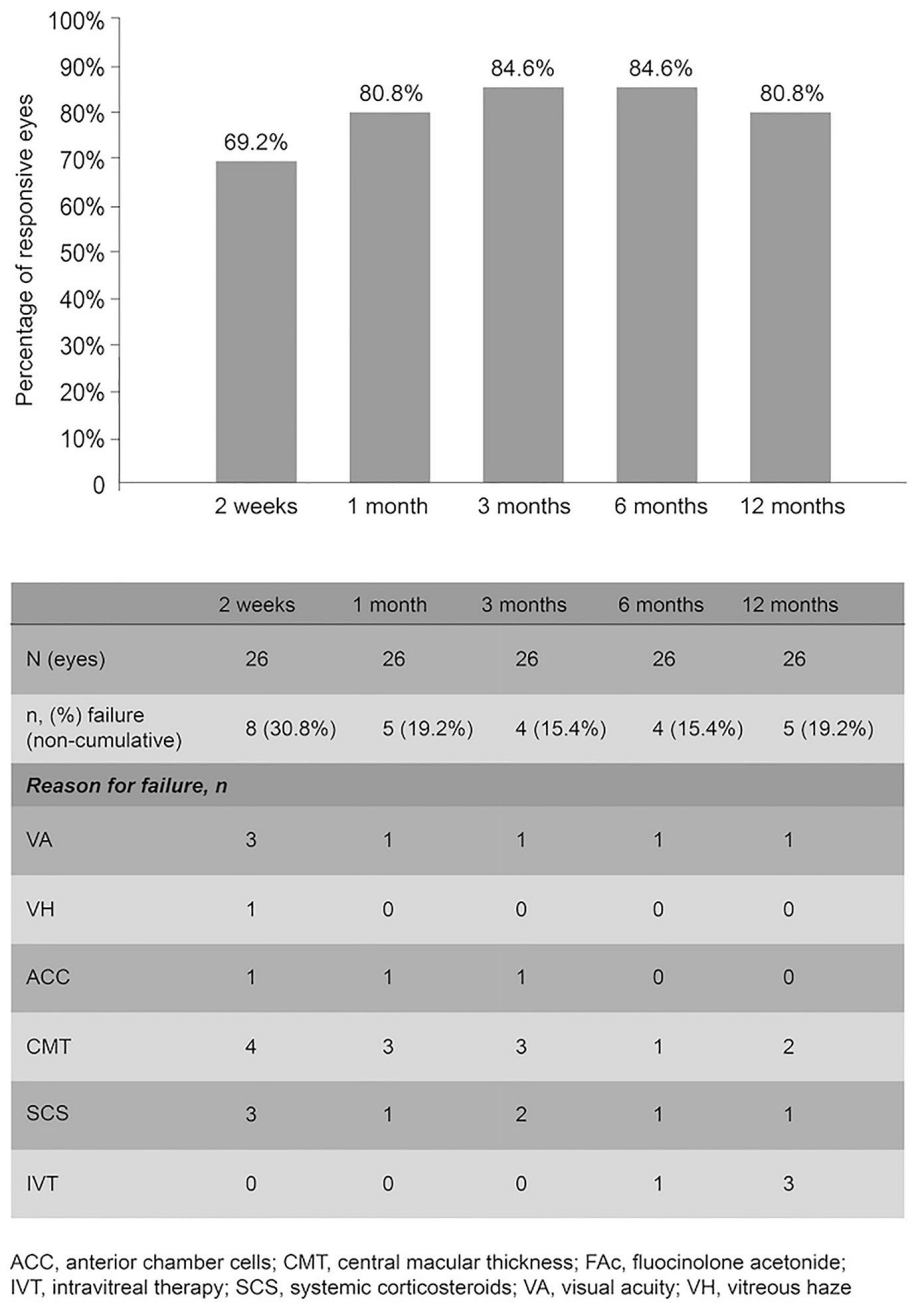
Effectiveness of the FAc implant assessed by multicomponent outcome measure at 2 weeks to 12 months after FAc implantation (*n* = 26)

As shown by the multicomponent endpoint, over the course of the 12-month study, the FAc implant was effective at every timepoint in 15 (57.7%) eyes, reaching peak effectiveness as soon as 2 weeks post-implantation. From month 1 onwards, 19 (73.1%) of eyes achieved effectiveness at every timepoint. The FAc implant was effective in a minimum of 69.2% (week 2) and maximum of 84.6% of eyes (months 3 and 6) (Fig. 1). The strategies trying to rescue an eye after the failure of FAc implant at a given time point were injecting a dexamethasone implant (one eye that failed at 6 and 12 months), anti-VEGF injection (two eyes that failed at 12 months) or by increasing systemic oral prednisone (one eye that failed at 3 months).

**Fig. 2:**
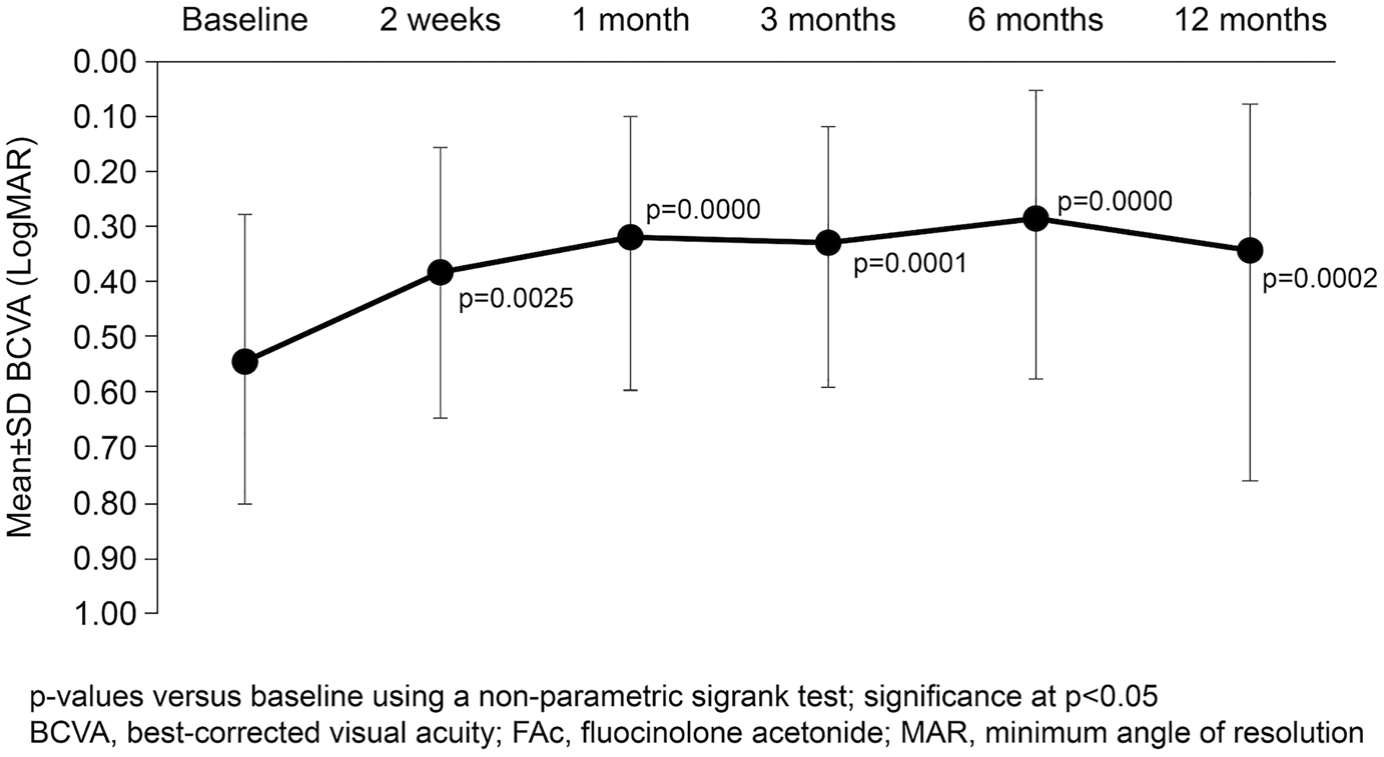
Mean BCVA (LogMAR) at baseline to 12 months after FAc implantation (*n* = 26)

**Fig. 3:**
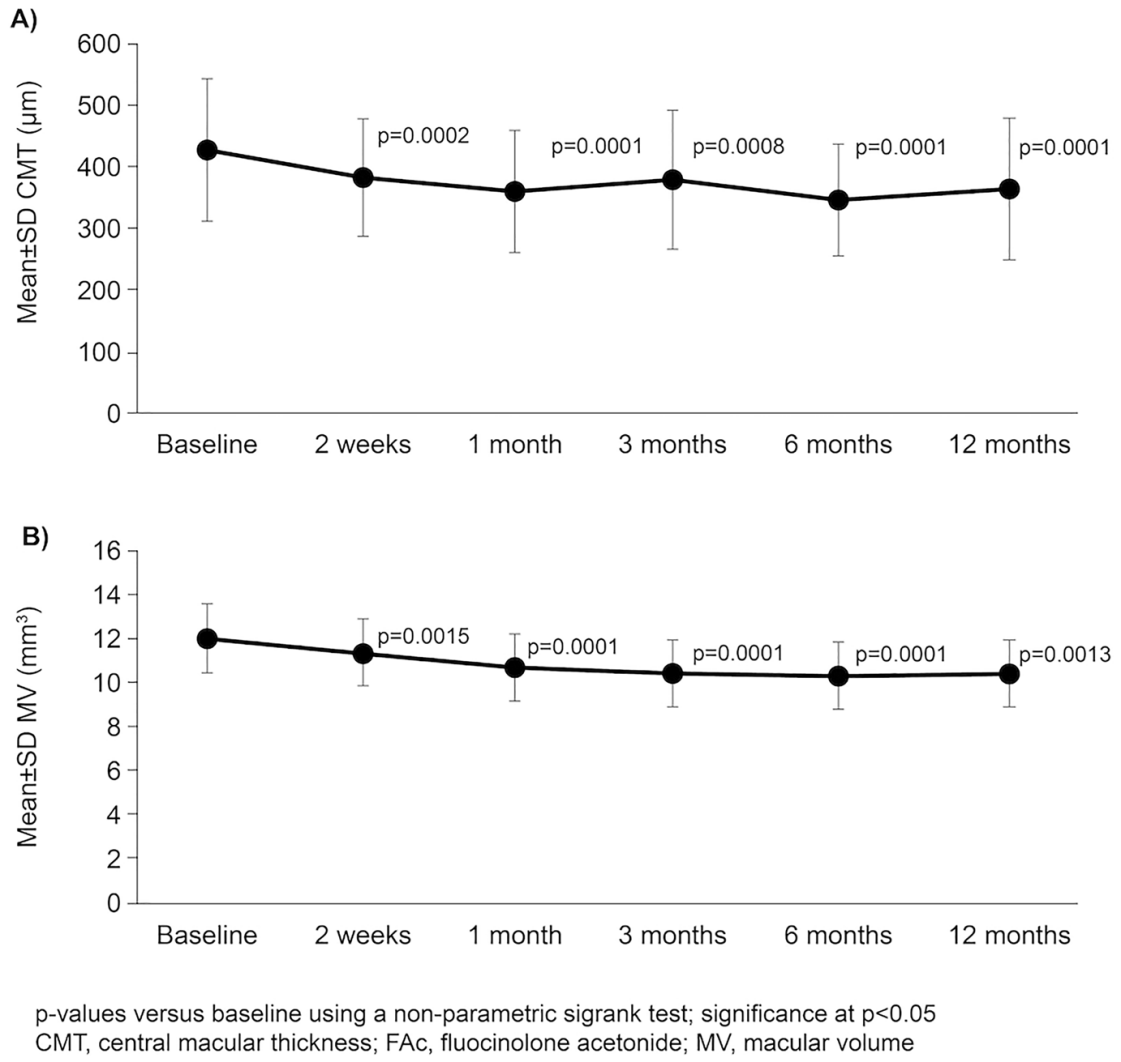
Mean CMT (**A**) and mean MV (**B**) at baseline to 12 months after FAc implantation (*n* = 26)

**Fig. 4:**
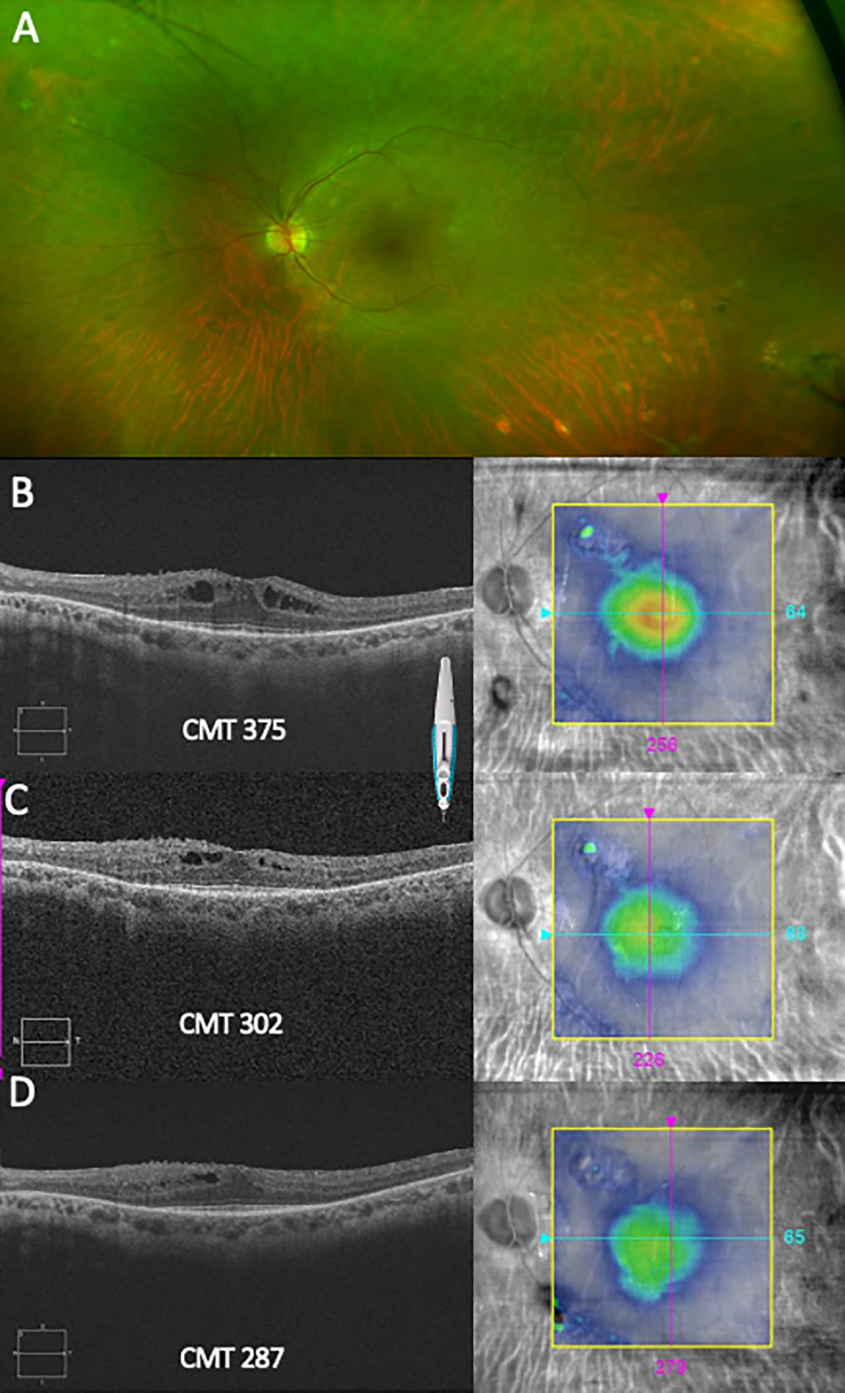
A 63-year-old female with sympathetic ophthalmia in the left eye secondary to a traumatism 17 years ago. She was refractory to multiple therapies (ciclosporin A, mycophenolate mofetil and tocilizumab) prior to initiating adalimumab; the last been also discontinued due to tongue cancer. Locally, five intravitreal dexamethasone implants (DEX) were necessary to resolve the uveitic macular oedema (UME). **A** Left eye Optomap® fundus shows diffuse yellowish–white choroidal lesions or Dalen–Fuchs nodules. **B** OCT B scan shows a recalcitrant UME after 5 DEX with a central macular thickness (CMT) of a 375 µm and a red central thickness map. **C** Improvement of the UME after 2 months of FAc implant is seen, with a CMT of 302 µm. **D** A resolved UME with a green central thickness map after 12 months of FAc implant. Outer external bands are preserved only in the central area of macular cube OCT, and visual acuity was 20/80 in all the visits in the follow-up

Mean BCVA (LogMAR) was significantly improved compared to baseline at all timepoints (all comparisons *p* < 0.01; Fig. 2). Mean CMT was significantly reduced vs. baseline at all timepoints (all comparisons *p* < 0.01; Fig. 3A), with the greatest reduction of 72.2 µm between baseline and week 2 reaching a maximum reduction of 105.5 µm at month 6. Similarly, significant reductions compared to baseline in mean MV were noted at all timepoints (all comparisons *p* < 0.01; Fig. 3B). A case report of a FAc implant is shown in Fig. 4.

**Fig. 5:**
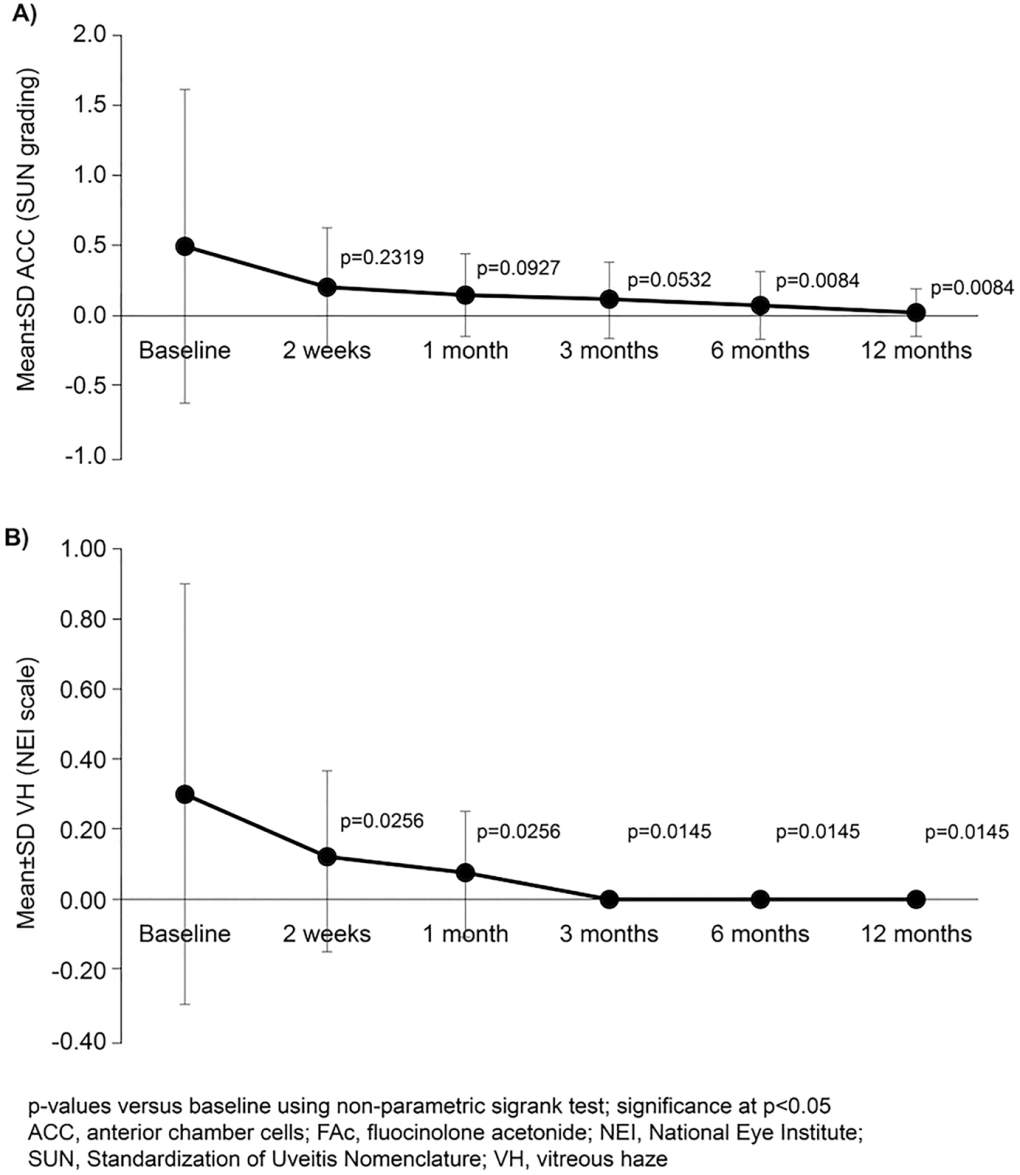
Mean ACCs (SUN grading; **A)** and VH (NEI scale; **B)** at baseline to 12 months after FAc implantation (*n* = 26)

In a categorical analysis, the percentage of patients with a preserved ellipsoid layer was found to be significantly greater than baseline (50.0%) at all timepoints from week 2 (*p* < 0.05), ranging from 73.1% (week 2) to 76.9% (all other timepoints; data not shown). As assessed using SUN grading, mean ACCs were reduced compared to baseline at all timepoints, with significance reached from month 6 to month 12 (*p* < 0.05; Fig. 5A). ACC ≥ 0.5 + decreased from 27% of the eyes at baseline to 23%, 23%, 19%, 11% and 8% at 2 weeks and months 1, 3, 6 and 12, respectively. Mean VH score was significantly reduced *vs*. baseline at all timepoints (*p* < 0.05; Fig. 5B). Mean ACCs results, VH ≥ 0.5 + decreased from 23% of the eyes at baseline to 15%, 11%, 0%, 0% and 0% at 2 weeks and months 1, 3, 6 and 12, respectively.

Eleven eyes (42.3%) failed, at least once, during the course of the study. The Kaplan–Meier curve until first failure has estimated a survival (initial efficacy) of 84.6% at 2 weeks, 69.2% at months 1, 3 and 6 and 57.7% at 12 months. However, 3 (11.5%) eyes did not meet efficacy criteria at 2 weeks, but reached efficacy at 1 month and was maintained up to month 12. Survival curve until first failure after 2 weeks post-FAc injection showed 96.2% survival at 1 month, 80.8% at 3 and 6 months, but declined to 69.2% at 12 months. Eight eyes (30.8%) failed, at least once, from 1 to 12 months of follow-up. Nevertheless, the survival analysis does not take into consideration that some eyes may fail at some timepoint and reach effectivity later, either spontaneously or after an adjuvant therapy.

**Fig. 6:**
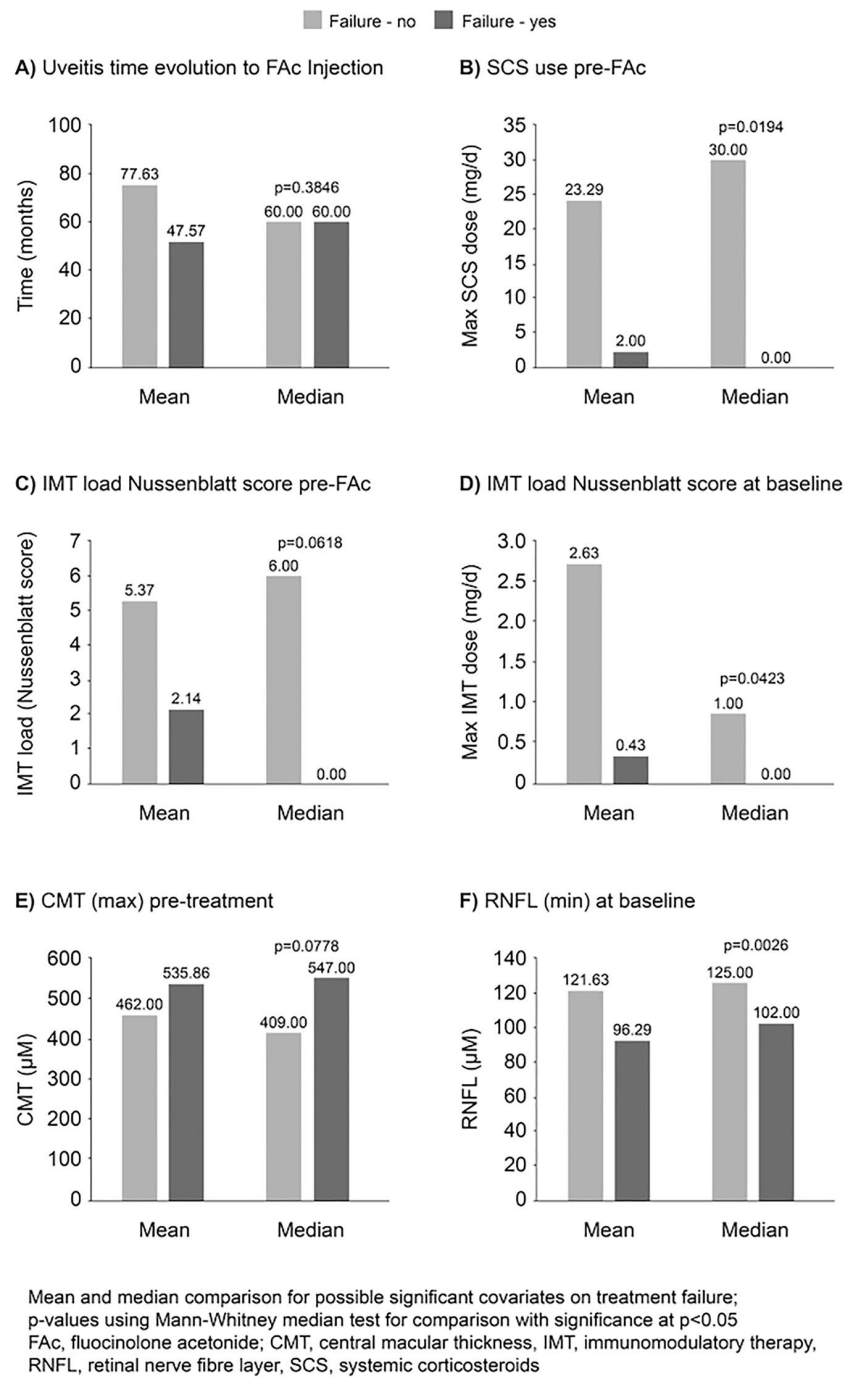
Univariate risk analysis of factors predicting treatment failure at 12 months after FAc implantation (*n* = 26)

In a univariate risk analysis at month 12, a higher systemic corticosteroid dose pre-FAc and IMT load (Nussenblatt score) at baseline and a thicker RNFL at baseline were found to be significant predictors of FAc implant efficacy (Fig. 6). Furthermore, using general estimating equations (GEE) modelling, factors predicting treatment failure were as follows: male patients, a thinner RNFL at baseline and ineffective treatment at 1 month (all *p* < 0.05). The aetiology of NIU was not a predictive factor for treatment failure.

### Safety

**Fig. 7:**
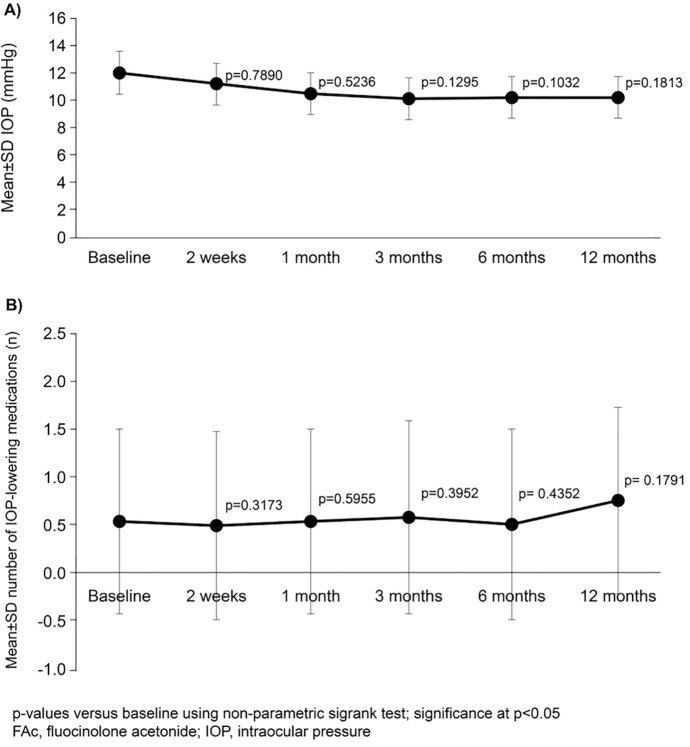
Mean IOP (**A**) and number of IOP-lowering medications (**B**) at baseline to 12 months after FAc implantation (*n* = 26)

There were no significant increases in IOP at any timepoint (Fig. 7A). IOP over 21 mmHg was recorded in 5 (19.2%) of the eyes at 2 weeks, in 3 (11.5%) eyes at 1, 3 and 6 months and in 2 (7.7%) eyes at 12 months. This IOP increase occurred in different eyes at each timepoint, and IOP was normalized by adding appropriate topical medication in all of them. No eye achieved IOP ≥ 30 mmHg at any timepoint. A tendency towards an increase in use of topical IOP-lowering medications was noted over the course of the study, but none of these changes reached significance (at any timepoint; Fig. 7B). The mean RNFL decreased significantly vs. baseline (114.81 µm) at every timepoint of the study, declining by 7.4% and 14.9% at months 6 and 12, respectively (all *p* < 0.001). Mean vertical cup/disc ratio measured by OCT significantly increased from baseline, with an increasing trend to month 6 (27.5% change *vs*. baseline) and falling slightly at month 12 (17.5% increase *vs*. baseline; all *p* < 0.01). After a case-by-case evaluation by a glaucoma specialist (M.P.), including visual field testing, from baseline to month 12, true glaucomatous progression or new glaucoma onset was not recorded. One eye showed less than 80 µm of RNFL at baseline, which was maintained to 12 months follow-up. Other adverse events reported were cataracts (*n* = 2), one of the transient post-injection subconjunctival haemorrhages (*n* = 1) and transient post-injection hypotony (*n* = 1).

### Additional therapies

**Fig. 8:**
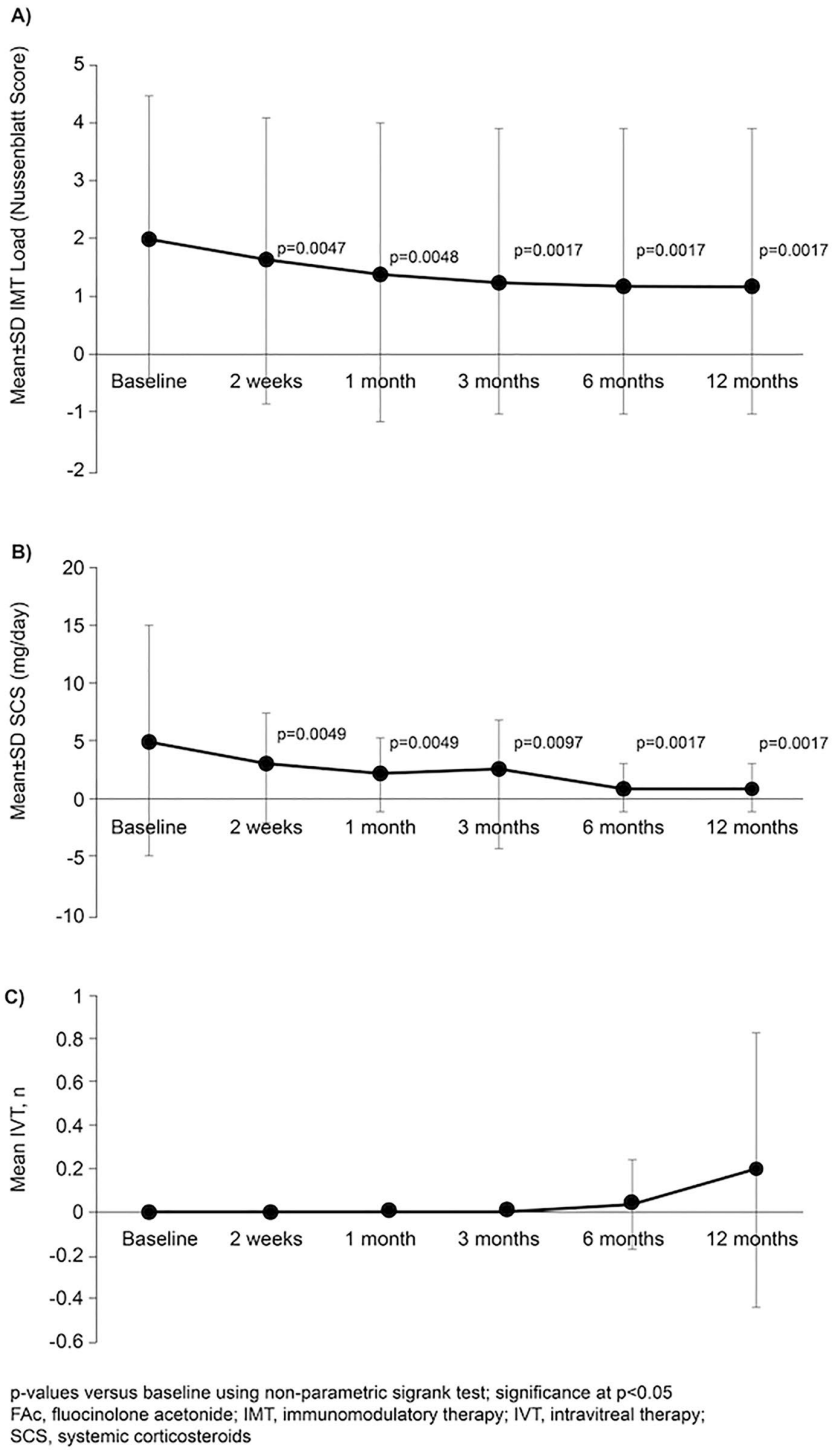
Mean IMT load (Nussenblatt score; **A**, SCS use (**B**) and number of IVT (**C**)) at baseline to 12 months after FAc implantation (*n* = 26)

Mean IMT load (Nussenblatt score) was significantly reduced *vs*. baseline at all timepoints (*p* < 0.05; Fig. 8A). Equally, systemic corticosteroid use over the course of the study was reduced (*p* < 0.05 at all timepoints; Fig. 8B). From week 2 to month 3, no patient received any adjuvant intravitreal injection; in contrast with month 6 and month 12, where intravitreal injections were needed in a mean of 0.04 injections per eye and 0.2 injections per eye, respectively (Fig. 8C).

## Discussion

Using a multicomponent outcome measure, this prospective study assessed the effectiveness of the 0.19-mg FAc implant while treating a series of NIU cases from November 2018 to November 2020 at a single uveitis unit in Spain (Clinic Institute of Ophthalmology, Barcelona, Spain). The composite endpoint demonstrated that the 0.19-mg FAc implant was effective in 15 (57.7%) eyes along the initial 12 months of the study, including an initial 2-week timepoint. However, effectiveness fluctuated between timepoints 16 (69.2%) eyes at week 2 to 22 (84.6%) eyes at months 3 and 6. Safety data showed a good tolerability and non-significant changes across a variety of measures (mean IOP, mean IOP-lowering medication use, mean RNFL and mean vertical cup/disc ratio). The multicomponent outcome measure used in this study covers a broad spectrum of outcomes, which were monitored in all patients along 12 months post-injection. In our study, we used nine outcome measures to assess treatment failure. Combined, these outcome measures provide a robust assessment of the effectiveness of the FAc implant such as inflammatory activity, therapeutic requirements, structural changes and functional outcomes. Indeed, given that these outcomes are frequently recorded in our routine clinical practice, such multicomponent outcome measure could provide a more complete picture of treatment effectiveness.

In the current study, we observed a rapid improvement in BCVA, which reached significance (*vs.* baseline) 2 weeks post-implantation and remained relatively stable over the following 12 months. A similarly rapid and sustained improvement was noted in the trial by Jaffe et al., where mean gain of ~ 4 and 7 EDTRS letters were observed after 1 and 3 months, respectively [21]. Our data are also st. in line with Battista et al. results, who report a steady and sustained improvement in BCVA with the FAc implant over 12 months; although, these results were only significant from month 6. However, the population included in the last had exclusively posterior uveitis with a mean ± SD duration of 8 ± 5 years (range 3–20). However, our study included patients with uveitis of any localization with a mean ± SD duration of 5.8 ± 4.9 (range: 0.5–18) years, indicating that earlier treatment may be beneficial [25].

Functional improvements were compared to structural outcomes measures. For instance, significant reductions compared with baseline (433.5 µm) in mean CMT were noted from as early as week 2 (-72.2 µm) and sustained to month 12 (-92.4 µm). This reduction is in agreement with the data from Jaffe et al. who observed an 82.5 µm reduction over 12 months from a baseline of 368.0 ± 145.0 µm [21]. The slightly greater decrease (~ 10 µm) in our study is probably due to higher CMT at the baseline. In the study by Studsgaard et al., mean CMT at 12 months following treatment with the FAc implant was reduced by 45 µm *vs.* baseline (314 μm [189–459 μm]). Again, the smaller reduction can likely be explained by a lower baseline CMT. Studsgaard et al. reported that they conventionally pre-treat patients in order to reduce the NIU recurrence rate prior administration of the FAc implant, this practice being possibly responsible for the lower baseline CMT [26].

Further, the percentage of patients with a preserved ellipsoid layer was significantly greater after FAc implantation at all timepoints. Integrity of the ellipsoid layer has been defined as a marker of better visual prognosis in UME with DEX implantation [27]. However, it is not clear whether cystoid spaces in UME may result in artefacts in the ellipsoid layer analysis, mimicking a loss of its integrity and recovering after UME resolution.

In the current study, following FAc implantation, measures of inflammation (ACC and VH) gradually declined over time, which supports the beneficial effect of the implant in controlling the underlying inflammation in NIU [21]. Furthermore, there is a marked absence of inflammatory relapses up to month 12; these data reflect that the FAc implant reduces NIU recurrences and though the underlying inflammation [21]. Throughout the course of our study, IMT and SCS dosage significantly decreased from week 2, which is a clinical manifestation of a low ocular inflammation. According to our study, FAc implant may help clinicians to reduce the burden of treatment on patients. Conversely, from month 6, there was a slight increase in the requirement for IVT.

The univariate risk analysis showed that a higher systemic corticosteroid dose pre-FAc, a higher IMT load at baseline and a thicker RNFL at baseline are significant predictors of FAc implant efficacy at month 12. GEE modelling demonstrated that the factors predicting treatment failure at month 12 were male patients, a thinner RNFL at baseline and ineffective treatment at month 1. Together, these data may identify subgroups of patients who may be more suitable for treatment with the FAc implant.

Mean IOP was stable throughout this study (i.e. change from baseline was not significant) which differs from other reports showing mean IOP increases with FAc implantation [21, 26, 28, 29]. For instance, Studsgaard et al. reported a mean IOP increase of 3 mmHg, with an absolute peak increase of 45 mmHg [26]. It is well known that inflammatory glaucoma benefits from low-dose corticosteroid therapy, which is able to better control a raised IOP, along with anti-hypertensive medications. In fact, a pivotal clinical trial of the FAc in uveitis with 36 months of follow-up found less risk of glaucoma surgery in FAc eyes versus simulated injection (sham). In our study, two patients who had previously received glaucoma surgery, IOP rise was negligible. These results are in agreement with a recent case report by Reddy et al. and the study by Studsgaard et al. (two eyes), in which past history of glaucoma surgery did not correspond to a higher rise in IOP [26, 30].

The RNFL decreased significantly at every timepoint. Uveitis has been described as a major confounding factor in assessing the thickness of the RNFL. Patients with active inflammation have a greater RNFL thickness due to swelling of the optic nerve. Moore et al. observed in 19 non-glaucomatous active uveitic eyes that the mean global and sectorial RNFL measurements were greater than the normative 95^th^ percentile. Moreover, in glaucomatous eyes with active or quiescent uveitis, the mean global RNFL was higher than the mean global RNFL reported in eyes with same stage of non-uveitic glaucoma [31]. Therefore, after successful control of inflammation, RNFL and other OCT measurements can be reduced as it occurred in our cohort, without meaning true glaucomatous progression. In these situations, or in cases of doubt, visual field test assessment can be a good alternative to monitor glaucomatous changes.

Regarding the aetiology of NIU in patients with systemic disease (i.e. Bechet’s disease, sarcoidosis, sympathetic ophthalmia, etc.), FAc implant should be considered as an adjuvant therapy for the management of ocular complications (i.e. UME) and should be continued under IMT as long as possible. On the other hand, FAc implant should be considered as a single therapy in patients with NIU not associated with systemic disease (i.e. Birdshot chorioretinopathy, punctate inner choroidopathy, IRVAN, etc.).

The current study encourages a novel broad-spectrum multicomponent tool as a reliable clinical predictor of FAc implant effectiveness. This measure covers structural, functional and inflammatory assessments (along with the need for additional treatments) as the basis for determining treatment failure. The study’s prospective design has permitted to tailor the study and to collect the data of interest. Further, the study was conducted in a real-world population who was reflective of care in the clinic setting. The study was limited by the relatively small number of eyes included (26 eyes); however, this is similar to some recent studies reported by Studsgaard et al. (22 eyes) and Battista et al. (10 eyes)[25, 26]. Also, the single-centre design may have interfere with the interpretation of the results in other countries/regions. For all these reasons, further investigations are needed to support the effectiveness of the 0.19-mg FAc in NIU.

## Conclusions

The 0.19-mg FAc implant is effective at 12 months of follow-up in the majority of patients treated for NIU in our study. A significant number of treated eyes reached a sustained functional and structural improvement from week 2 to month 12 after implantation as assessed by a novel multicomponent endpoint. No major safety concerns were raised during the course of the study at 12 months follow-up.
